# Fish Pedicure: Review of Its Current Dermatology Applications

**DOI:** 10.7759/cureus.8936

**Published:** 2020-06-30

**Authors:** Terri Shih, Samiya Khan, Shawn Shih, Amor Khachemoune

**Affiliations:** 1 Dermatology, David Geffen School of Medicine at University of California Los Angeles, Los Angeles, USA; 2 Dermatology, University of Texas Health Science Center at San Antonio, San Antonio, USA; 3 Dermatology, University of Central Florida College of Medicine, Orlando, USA; 4 Dermatology, State University of New York Downstate Medical Center, Brooklyn, USA

**Keywords:** psoriasis, fishes, complementary medicine, crypinidae

## Abstract

Ichthyotherapy or fish pedicure is a unique form of biotherapy in which the species *Garra rufa* or doctor fish is used to exfoliate the skin and potentially aid in healing diseases, such as psoriasis. The practice has gained popularity since its origins in Kangal Fish Spring in Turkey; however, safety concerns, especially among immunocompromised patients, remain. This article reviews the studied dermatological benefits of ichthyotherapy and theorized mechanisms of action. Included are cases examining both infectious and noninfectious complications of this procedure. This review highlights the need to educate susceptible patients about possible adverse effects and the need for more studies assessing this procedure.

## Introduction and background

**Figure 1:**
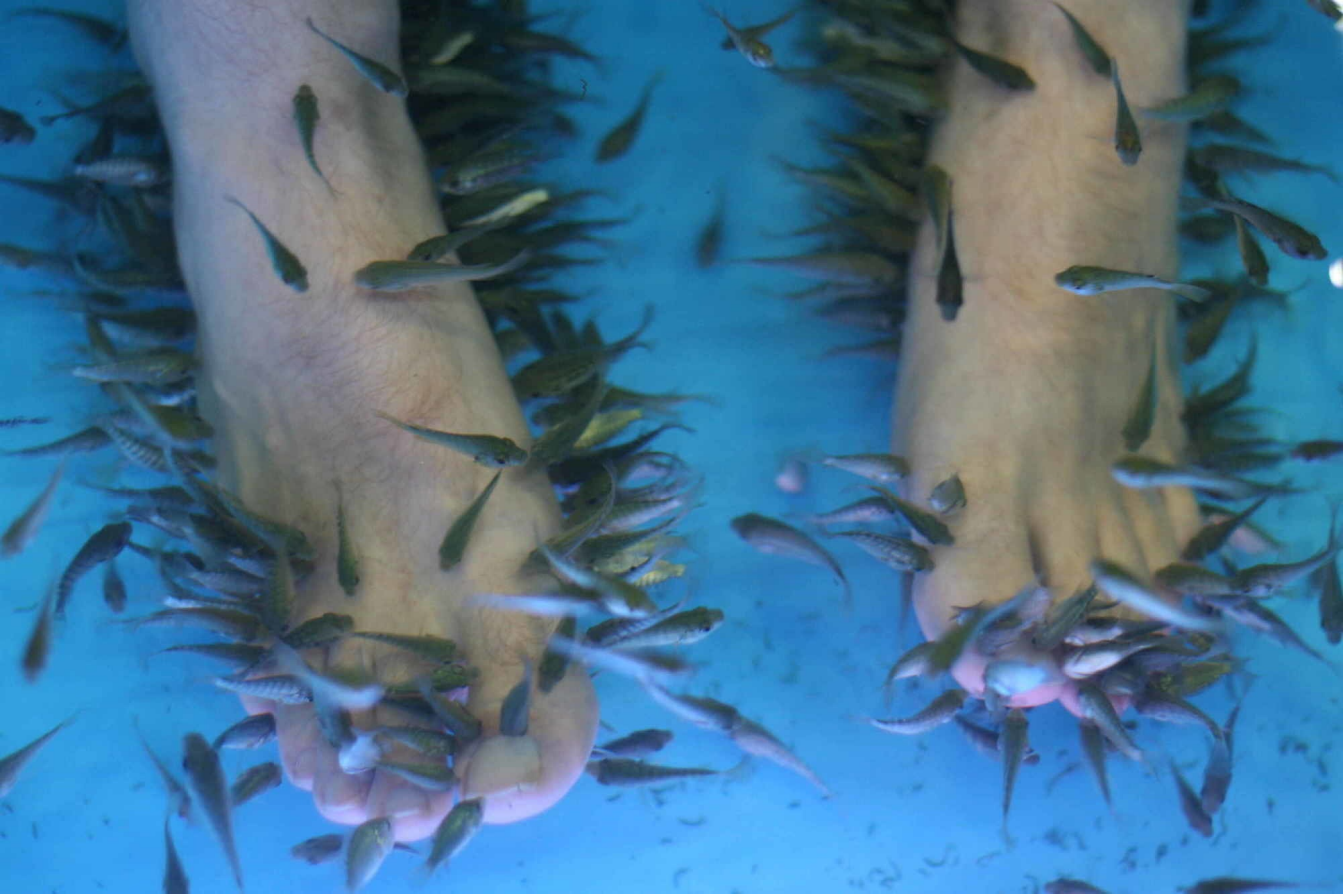
Fish Pedicure Procedure Fish Spa. (2008): https://www.flickr.com/photos/geoff_leeming/2723249699

Ichthyotherapy, also known as fish pedicure or fish spa, is a form of biotherapy in which the hands, feet, and whole body are immersed in a pool or tub of water filled with the fish *Garra rufa* which feed on dead human skin (Figure 1). *G. rufa*, also known as doctor fish, is a small species of cyprinid native to the Anatolia and West Asian regions. The species is omnivorous and normally feeds on phytoplankton or zooplankton but can feed on dead human skin when food is scarce.

Ichthyotherapy originated in Kangal Fish Spring in Turkey, a spring located in Kangal, a district of the Sivas province. According to local history, the therapeutic properties of the spring were first noticed in the early 1900s by a shepherd who healed his injured feet in the spring water. This led to the development of public pools and lodging facilities near the spring in the 1960s [1]. Today the Kangal Spring is designated as a certified thermal health center by the Turkish Ministry of Health and offers a 21-day treatment for psoriasis for visitors from around the world. This therapy provides an alternative to other forms of biotherapies which have been shown to improve psoriasis such as balneotherapy and thalassotherapy or treatment with seawater and mineral water, respectively [2,3]. Anecdotally, ichthyotherapy can also be used to treat skin diseases other than psoriasis, such as acne and eczema [1]. Due to the high temperature of the water in the Kangal thermal spring, plankton is scarce, making dead human skin an easily acquirable food source. There is another species of cyprinid fish found in Kangal Fish Spring named *Cyprinion macrostomus* that also belongs to the Cyprinidae family and plays the same role [4]. However, *G. rufa* is the main species imported by countries worldwide as the popularity of ichthyotherapy continues to soar, particularly in the form of fish pedicure (involving only the feet). Due to overharvesting, *G. rufa* has been given legal protection in Turkey, with most of the fish now coming from suppliers in the Far East [5]. While the term ‘ichthyotherapy’ has been used to describe other fish-related therapeutic modalities unrelated to fish spa, we will use this term here to exclusively describe treatment using *G. rufa*.

With this review, we hope to clarify the benefits and complications associated with ichthyotherapy found in the literature in order to dispel any myths concerning this unique treatment.

## Review

Studied dermatological indications

To our knowledge, Ozcelik et al. published the first study on the therapeutic effect of ichthyotherapy, specifically at Kangal Fish Spring, on psoriasis [6]. A total of 87 psoriasis patients were recruited and used the pool twice daily with a mean length of stay of 11.6 days and a mean duration of each session of 7.4 hours, as freely determined by patients. The effect of ichthyotherapy on the participants’ Psoriasis Area and Severity Index (PASI) scores over the treatment period was studied with follow-ups every three days. A total of 65 (74.7%) patients had plaque-type psoriasis. Of the study group, 52 (59.7%) came to the hot spring for the first time and 35 (40.3%) had come to hot spring previously. The median PASI score decreased from 9.6 at the first examination to 0 at 21-day follow-up, although only 14 patients remained in the study by the 21-day follow-up for reasons not stated. While 35 patients reported having significantly longer remission periods after ichthyotherapy when compared to topical corticosteroid therapy, it is unclear how these results were obtained.

Grassberger et al. conducted a retrospective pilot study in Austria to evaluate the efficacy and safety of ichthyotherapy in combination with ultraviolet A (UVA) radiation for treating psoriasis [7]. A total of 67 patients took two-hour baths followed by three to five minutes of UVA therapy daily for three weeks. Overall there was a 71.7% reduction in PASI score compared to baseline, with 31 (46.3%) achieving PASI-75 and 61 patients (91%) achieving at least PASI-50. Patient-reported outcomes showed 87.5% of the patients having more favorable outcomes with ichthyotherapy compared with other therapies and 61.5% having less severe relapses after ichthyotherapy.

In both studies, ichthyotherapy treatments were not limited to the lower extremities as patients took full-body baths with the fish. However, treatment protocols differed between Ozcelik and Grassberger. For example, in the study by Grassberger et al., each patient was allocated a personal bathing tub that was constantly filtered and sterilized, whereas, in the Kangal Springs, each patient was required to take baths with 10-20 other patients simultaneously. Moreover, approximately 250-400 fish were used in each tub in the study by Grassberger et al; however, there was no estimate of the number of fish used in the study by Ozcelik and colleagues. Therefore, standardized protocols are needed. 

Ichthyotherapy is commonly used as a treatment for plantar hyperkeratosis and has been anecdotally reported to treat rheumatological and neurological disorders [1]. No clear evidence has been found to support these claims. 

Mechanism

While the exact mechanism of action of ichthyotherapy is not completely understood, the fish may facilitate penetration of UV light by clearing the psoriatic scales as it strikes and licks the skin [2]. Reverse Koebner phenomenon may also explain the therapeutic effect of removing the scales which result in superficial ulcerations [8]. In contrast to the Koebner phenomenon in which new lesions related to an existing dermatosis appear at sites of trauma, the reverse Koebner phenomenon, first described among psoriasis patients, is defined by the disappearance of such lesions after trauma [9]. These two phenomena are mutually exclusive, and a study by Eyre et al. found that pre-existing disease activity did not predict a positive Koebner reaction after trauma among plaque psoriasis patients [8]. 

Some claims state that the fish secretes a natural enzyme in their saliva called diathanol that helps new skin grow, though this remains largely unproven and may be a marketing ploy [5]. It is also thought that the high selenium concentration of the Kangal Spring water may be contributory since psoriasis patients often have low levels of this mineral. However, ichthyotherapy appears to work even in low concentrations of selenium; thus, the role of this element remains debatable [2]. While selenium is known to have antioxidative and anti-inflammatory properties, its supplementation has not been shown to improve psoriasis disease activity [10,11]. However, Ozcelik et al. argued that the fish spa at Kangal hot spring should not be considered as ichthyotherapy, but rather a form of balneotherapy or the use of thermal mineral water to heal disease. He argues that the use of fish to remove squamae mainly serves to enhance the absorption of antioxidants and immunomodulatory minerals, which leads to improvement in a patient’s disease [6].

Complications of ichthyotherapy

Despite these benefits, various infectious and noninfectious concerns exist in regard to the safety of ichthyotherapy. If the patient has an underlying illness with reduced immunity or open skin lesions, the potential risk of infection is increased and therefore such patients should be cautioned against undergoing this treatment [12].

Infectious Complications

The major concern of ichthyotherapy is the risk of infection as the same water and fish are frequently used for subsequent clients [13]. During this procedure, pathogens can potentially be transmitted from fish to persons (termed zoonotic disease), from water to person through contaminated water, or from person to person via the equipment or fish. While disinfecting the water would reduce the likelihood of infection, disinfection is often not possible or routinely performed because many conventional water disinfectants also kill the fish [14]. In addition, although the water in fish spas is commonly filtered, most applied filters do not remove microorganisms, especially those in biofilms or on fish skin [12]. 

The transport of fish in stressful, crowded conditions may lead to poor fish health and create an environment favorable for infection of the fish. An outbreak of S*treptococcus agalactiae* infection was found in 6,000 *G. rufa* fish from Indonesia imported to the United Kingdom (UK) in 2011, in which 95% of the fish died while the remaining fish were euthanized [15]. Bacterial strains isolated from *G. rufa* imported from Indonesia into the UK in 2011 included *Aeromonas *spp, *Vibrio vulnificus*, *Vibrio cholerae*, *Mycobacteria *spp., and *S. agalactiae*, all of which demonstrated multidrug resistance. Similar bacterial strains in addition to *Pseudomonas aeruginosa* were isolated in fish spas in the Netherlands [12]. In another study, *S. agalactiae* was isolated from a batch of *G. rufa *from a fish spa in Ireland [16]. A more recent study by Volpe and colleagues looking at the cause of two mass mortality outbreaks among fish pedicure spas in Italy found the presence of multiple zoonotic bacterial species, such as *Aeromonas veronii*, *Aeromonas hydrophila*, *V. cholerae,* *Shewanella putrefaciens*, *Mycobacterium marinum*, and *Mycobacterium goodii *among both sick and asymptomatic fish due to poor handling and environmental conditions [13]. This study highlights the first detection of *M. goodii*, an emerging nosocomial human pathogen, among fish or any animal, and therefore a potential zoonotic reservoir.

While various pathogenic bacteria have been found in association with *G. rufa*, the species of fish used for ichthyotherapy, no reports exist of any viral, fungal, protozoal, or helminth pathogens in association with this species. 

The aforementioned bacteria can cause a variety of illnesses among humans, and species such as *V. vulnificus*, *M. marinum*, *P. aeruginosa*, *Staphylococcus aureus*, and *S. agalactiae* are well known to cause cutaneous infection in humans. Four case reports of a cutaneous infection following fish pedicure exist in the literature (Table 1). For example, a case of methicillin-resistant *S. aureus* infection attributed to ichthyotherapy was reported in a healthy patient six days after ichthyotherapy in Spain. The patient was successfully treated after two weeks of cotrimoxazole and rifampin [14]. Another report of a cutaneous infection attributed to ichthyotherapy was reported in a patient with *S. aureus* infection of bilateral feet three days after ichthyotherapy in Greece. The patient was successfully treated after five days of 0.05% sodium hypochlorite footbaths and oral ciprofloxacin (500 mg/day) [17]. In 2015, Vanhooteghem et al. reported a case of fish pedicure-induced *Aeromonas sobria* superficial necrotic bullous dermatitis in a 64-year-old patient with previously undiagnosed type II diabetes, successfully treated with three weeks of oral ciprofloxacin [18]. The patient’s diabetes remained undiagnosed until the patient was prompted by the new rash to seek medical care, possibly because he had not had routine checkups with any physician. Nonetheless, underlying diabetes likely predisposed the patient to infection, induced by the fish pedicure in this case. In a different paper, Vanhooteghem et al. also described an ichthyotherapy-induced mycobacterial infection that occurred in a patient on the dorsum of the left foot after a public ichthyotherapy procedure in Bangkok [19]. *M. marinum* was found to be the causative agent. The patient was diagnosed with aquarium granuloma, also known as fish tank granuloma, and successfully treated after three months of oral rifampicin and clarithromycin.

**Table 1:** A Summary of Case Reports of Cutaneous Infection Following Ichthyotherapy

Author	Causative Pathogen	Clinical Presentation	Treatment	Outcome
Sugimoto and colleagues [14]	Staphylococcus aureus	Cellulitis of the right forefoot, interdigital macerations due to tinea pedis, focal abscess in the third toe	Incision and drainage of the abscess. Cotrimoxazole and rifampin for two weeks	Full recovery after two weeks of treatment
Veraldi and colleagues [17]	Staphylococcus aureus	Dermatitis on both feet	0.05% sodium hypochlorite footbaths. Oral ciprofloxacin (500 mg/day for 10 days)	Remission after five days of therapy
Vanhooteghem and colleagues [18]	Aeromonas sobria	Superficial necrotic dermatitis of the right tibia	Oral ciprofloxacin 500 mg twice daily. Local gentamicin cream	Full recovery after four weeks of treatment
Vanhooteghem and Theate [19]	Mycobacterium marinum	Erythematous plaque on the left foot	Clarithromycin (1 mg/day) for three days and oral rifampicin (750 mg/day)	Nodules regressed after eight weeks of therapy

Theoretically, bloodborne pathogens, such as hepatitis B virus or human immunodeficiency virus, could be transmitted from ichthyotherapy if blood from a small cut spills into the tank of one user and infects an open cut in a subsequent user of the same fish tank. However, no reports of this phenomenon exist, making this possibility unlikely. *G. rufa* has only been documented to feed on human skin (and not blood like mosquitos), further reducing the likelihood of bloodborne virus transmission. 

Noninfectious Complications

In addition to cutaneous infections, onychomadesis, also known as defluvium unguium, was reported in a patient who had ichthyotherapy a few months prior to the onset of her toenail abnormalities, likely due to trauma from fish biting multiple nail units [20]. While onychomadesis is associated with severe systemic diseases, infection, trauma, and drugs, the patient’s medical history was negative for these factors, suggesting that the fish pedicure she had had a few months prior was the causative agent. Mild temporary bleeding from open crusted lesions was also reported as a side effect to ichthyotherapy in a patient with eczema in a study by Grassberger and Hoch; therefore, mild trauma may be an additional side effect [7]. 

To date, no other noninfectious complications have been reported with ichthyotherapy.

Discussion

While the two studies on ichthyotherapy reported favorable results for the treatment of psoriasis, neither were randomized controlled studies. While Ozcelik et al. reported a reduction of median PASI to zero by the 21-day follow-up, only 16% of the patients remained in the study [6]. It is unclear whether patients dropped out for lack of efficacy or for other reasons. Grassberger reported an overall 71.7% reduction in PASI score compared to baseline after treatment with UVA and ichthyotherapy for three weeks, with 91% of the patients achieving at least 50% reduction. Although 87.5% of the patients having more favorable outcomes with ichthyotherapy compared with other therapies and 61.5% reported having less severe relapses after ichthyotherapy, the response rate for the patient-reported outcome questionnaire was only 60% [7]. Furthermore, the reduction in PASI may be less significant in ichthyotherapy monotherapy without the UVA component. More research is also needed to determine whether ichthyotherapy treatments require water mineral composition that mirrors that of Kangal spring water to achieve similar therapeutic efficacy.

While the risk of bacterial infection associated with ichthyotherapy appears to be very low, it is important for patients to be aware of such risk. Those who are immunocompromised or have a break in their skin, in particular, should avoid ichthyotherapy due to infection risk. Fish pedicure is banned in many parts of the US, Canada, and Europe due to health concerns and condemned by animal rights activists due to similar health concerns and lack of concern for animal welfare [5]. If ichthyotherapy can be provided by treatment facilities in a controlled environment with proper sanitation measures, it may become more accepted by patients and the general population. For example, medical maggot therapy, which is a form of biotherapy, like ichthyotherapy, is approved by the Federal Drug Administration for wound debridement and has been used for centuries for wound care [21]. Unfortunately, with ichthyotherapy, the fish and the pool cannot be disinfected between uses without potentially harming the fish. Lastly, more evidence is needed to confirm the association between ichthyotherapy and onychomadesis since only there has only been one reported case, though any form of trauma can theoretically induce onychomadesis.

Further research is warranted to compare ichthyotherapy with other treatment modalities, such as topical steroids and systemic/immunomodulating agents, to determine its relative efficacy, when used alone and/or combined with other therapies. Aside from psoriasis, the potential role of ichthyotherapy to treat other dermatological conditions, such as plantar hyperkeratosis, should be investigated. In addition to ichthyotherapy, other forms of alternative therapies, such as balneotherapy and thalassotherapy, may also be worth exploring for the treatment of psoriasis and other cutaneous diseases.

## Conclusions

Currently, there is no strong evidence to support the routine use of ichthyotherapy for psoriasis or other cutaneous diseases. While the risk of infectious complications appears to be very low, it should not be overlooked, especially among immunocompromised patients. However, it may be premature to fully dismiss this unique treatment modality as a potentially cost-effective treatment option without any known systemic side effects. Prospective randomized controlled studies are warranted to better establish the therapeutic efficacy and side effect profile of ichthyotherapy for treating psoriasis and potentially other skin diseases.
